# A modified lethal–potentially lethal model for oxygen‐mediated FLASH sparing in stem cell niches

**DOI:** 10.1002/mp.70469

**Published:** 2026-05-10

**Authors:** Sumin Zhou

**Affiliations:** ^1^ Department of Radiation Oncology University of Nebraska Medical Center Omaha Nebraska USA

**Keywords:** FLASH radiotherapy, LPL model, nuclear oxygen depletion, oxygen enhancement ratio, stem cell niche

## Abstract

**Background:**

Ultra‐high dose rate (FLASH) irradiation can reduce normal‐tissue toxicity while preserving tumor control, but a mechanistic explanation consistent with classical radiobiology remains incomplete. In particular, oxygen‐depletion arguments based solely on bulk tissue oxygenation can appear inconsistent with clinically relevant fraction sizes, motivating a DNA‐target‐level oxygen formulation.

**Purpose:**

To develop a theory‐based mechanistic extension of the Lethal and Potentially Lethal (LPL) model that explains oxygen‐mediated FLASH trends without prescribing dose rate‐dependent radiosensitivity, and to identify the baseline nuclear oxygen window in which sparing is expected to be largest.

**Methods:**

We introduce an explicit Precursor Lesion population whose fate is governed by competing chemical restitution/repair versus oxygen‐dependent fixation into potentially lethal and lethal lesion channels. Fixation kinetics are coupled to a time‐varying nuclear oxygen tension, $pO_{2}\left(t\right)$, which decreases via radiolytic depletion during irradiation and recovers toward a baseline via reduced‐order reoxygenation kinetics. To address the oxygen paradox, we distinguish bulk vascular oxygenation from a lower effective DNA target‐level oxygenation that may arise in regulated stem‐cell niches because of niche hypoxia and intracellular oxygen consumption. Oxygen modulation is implemented through a mechanistic exponential OER formulation parameterized by an oxygen‐fixation rate constant, while retaining classical LPL behavior in the conventional low‐dose‐rate limit.

**Results:**

The model predicts that oxygen‐mediated FLASH sparing is largest when baseline nuclear oxygenation lies in an intermediate physiologic‐hypoxia regime, corresponding to the steep oxygen‐responsive portion of the OER curve. In the reference parameter set, a quiescent normal‐tissue niche with baseline $pO_{2,0}$ = 3 mmHg shows appreciable sparing under FLASH delivery, whereas sparing is minimal when the baseline lies near either the OER floor ($pO_{2,0}$ = 0.2 mmHg) or the OER saturation plateau ($pO_{2,0}$ = 30 mmHg). Sensitivity analyses preserve this intermediate oxygen window while shifting the magnitude and threshold of the effect.

**Conclusions:**

By explicitly resolving Precursor Lesion fixation kinetics and by treating niche‐to‐nucleus oxygenation as an effective target‐level variable, this mechanistic LPL framework predicts that oxygen‐mediated FLASH sparing is most likely when baseline oxygenation lies within an intermediate physiologic‐hypoxia window. The model should therefore be viewed as a mechanistic, testable framework rather than as a universal explanation of all FLASH responses.

## 1 Introduction

Radiation‐induced cell killing reflects processes spanning many orders of magnitude in time, from energy deposition and radical chemistry to the fixation and repair of DNA damage.^1^, ^2^ To enable quantitative prediction and treatment planning, classical radiobiological formalisms such as the Linear‐Quadratic (LQ) model and the Lethal and Potentially Lethal (LPL) model collapse these multiscale processes into effective kinetic parameters that are valid under conventional clinical delivery conditions.^3^, ^4^

Ultra‐high dose rate (FLASH) irradiation, typically delivered in sub‐second pulses at dose rates several orders of magnitude above conventional radiotherapy, has challenged these reduced descriptions. Multiple preclinical studies report reduced normal‐tissue toxicity for a given dose while maintaining tumor control, motivating substantial interest in clinical translation.^5^, ^6^ Consistent with this trajectory, the first‐in‐human FLASH radiotherapy treatment demonstrated feasibility and favorable early safety outcomes.^7^, ^8^

Despite rapid experimental progress, a unifying mechanistic explanation remains elusive. Recent reviews and perspective pieces continue to highlight the complexity of competing mechanisms, including oxygen depletion, stem‐cell niche hypotheses, and immune modulation.^9^, ^10^, ^11^ Many modeling approaches reproduce dose rate effects by prescribing dose rate‐dependent radiosensitivity or by imposing empirical modifications of oxygen enhancement based on bulk tissue oxygenation.^12^ In parallel, several mechanistic hypotheses have been advanced, including radiolytic oxygen depletion (ROD) models that propagate transient oxygen loss into changes in the oxygen enhancement ratio (OER),^13^, ^14^, ^15^ as well as physico‐chemical and redox‐metabolic frameworks emphasizing radical recombination kinetics and differential oxidative stress handling in normal versus tumor tissue.^16^, ^17^

Oxygen is central to these hypotheses because it increases radiation damage by chemically fixing otherwise repairable lesions.^18^, ^19^ The time window for oxygen to modify radiation injury can be on the order of tens of milliseconds, consistent with classic pulsed‐irradiation studies and mechanistic oxygen‐fixation modeling.^20^, ^21^ Accordingly, ROD during ultra‐rapid dose delivery provides a plausible pathway to reduced fixation and increased apparent radioresistance.

However, an apparent paradox persists. When oxygen depletion is analyzed using bulk extracellular oxygen tensions, the predicted dose required to drive tissue into a low‐OER regime can exceed typical FLASH fraction sizes.^14^ Moreover, ROD models predict that dose‐rate dependence should be strongest only within an intermediate oxygen window, with limited effect at both high and very low baseline oxygen levels.^13^, ^14^ These observations suggest that the relevant oxygen variable may not be the bulk vascular partial pressure but a more localized target‐level quantity at the DNA site. Therefore, we treat the DNA target‐level oxygen tension as the state variable governing fixation kinetics rather than bulk vascular $pO_{2}$.

We propose that one biologically relevant target of normal‐tissue injury—particularly in tissues whose acute epithelial maintenance or later functional recovery depends on spatially organized regenerative compartments—is the stem/progenitor cell niche. Many tissues in which FLASH sparing is reproducibly reported (e.g., skin, intestine, hematopoietic system, and brain) are maintained by compartments whose stem cells reside in physiologic hypoxia to preserve quiescence and genomic integrity.^22^, ^23^, ^24^, ^25^, ^26^, ^27^, ^28^ This represents a controlled, functional state essential for stem‐cell maintenance, distinct from the unregulated pathological anoxia found in necrotic tumor cores. Direct in vivo measurements support markedly reduced oxygen tensions in bone marrow microenvironments,^29^ and the concept of physiological normoxia emphasizes that many functional cellular compartments operate well below atmospheric oxygen levels.^30^

Consistent with the conceptual suggestion by Pratx and Kapp that hypoxic normal stem‐cell niches may be especially relevant to FLASH,^13^ we develop a mechanistic modified LPL (M‐LPL) framework that makes the oxygen‐dependent fixation step explicit through an additional Precursor Lesion state. Oxygen dependence enters only through the time history of the nuclear oxygen partial pressure $pO_{2}\left(t\right)$, coupled to radiolytic depletion and reduced‐order recovery toward a baseline value. The model is constructed to recover classical LPL behavior in the low‐dose‐rate limit,^3^ while enabling prediction of dose‐rate and pulse‐structure effects through physically interpretable oxygen and time‐scale parameters.

By distinguishing target‐level nuclear oxygenation in stem/progenitor niches from bulk vascular oxygenation, the framework provides a testable explanation for one possible route to normal‐tissue sparing at ultra‐high dose rates. At the same time, it does not presume that oxygen‐mediated fixation is the only FLASH mechanism or that the same compartmental logic applies uniformly across all tumor models.^14^, ^31^

## 2 METHODS

This section summarizes the model components used to connect dose rate delivery to oxygen‐mediated lesion fixation: (i) the classical LPL lesion‐kinetic formulation, (ii) a reduced‐order description of target‐level oxygen dynamics during irradiation, (iii) a niche‐to‐nucleus oxygen gradient parameterization for normal‐tissue stem cell compartments, (iv) a modified LPL system with an explicit Precursor Lesion state, and (v) the numerical strategy for solving the stiff kinetic system. Throughout, $pO_{2}\left(t\right)$ denotes the nuclear (DNA target–level) oxygen partial pressure.

### 2.1 Classical LPL formulation

**FIGURE 1:**
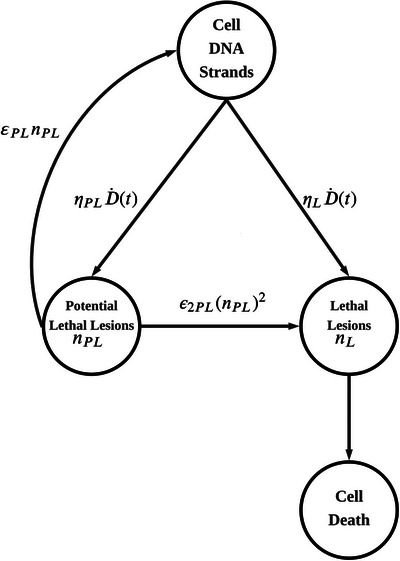
Flowchart of the classical Lethal and Potentially Lethal (LPL) model. Radiation induces two distinct classes of damage: potentially lethal lesions ($n_{PL}$) and lethal lesions ($n_{L}$), with yields $\eta_{PL}$ and $\eta_{L}$, respectively. The fate of $n_{PL}$ is governed by a competition between first‐order correct repair ($\varepsilon_{PL}$) and second‐order binary misrepair ($\varepsilon_{2PL}$), the latter of which contributes to the lethal lesion pool.

The classical LPL model describes radiation‐induced cell killing through the coupled evolution of potentially lethal lesions, $n_{PL}$, and lethal lesions, $n_{L}$ (See Figure 1). In its standard form, these ensemble‐averaged populations obey:^3^

(1a) $$\frac{d}{dt}\;n_{PL}=\eta_{PL}\;\dot{D}\left(t\right)-\varepsilon_{PL}n_{PL}-\varepsilon_{2PL}{\left(n_{PL}\right)}^{2}$$

(1b) $$\frac{d}{dt}\;n_{L}=\eta_{L}\;\dot{D}\left(t\right)+\varepsilon_{2PL}{\left(n_{PL}\right)}^{2}$$

Here $\dot{D}\left(t\right)$ denotes the instantaneous dose rate (Gy/s). PL and L stand for Potentially Lethal and Lethal in the Equations (1a) and (1b). Among the four model parameters, $\eta_{PL}$ and $\eta_{L}$ are potentially sensitive to environmental conditions, for example, $pO_{2}$, while $\varepsilon_{PL}$ and $\varepsilon_{2PL}$ are much more robust constants.

The reference values for the four model parameters ($\eta_{PL}$, $\eta_{L}$, $\varepsilon_{PL}$, and $\varepsilon_{2PL}$) used in our numerical simulations were taken from Curtis's analysis of the Cs‑137 irradiation study of the C3H10T1/2 cell line^3^, ^32^ and are listed in Table A1. The parameter $\eta_{L}$ is defined as the reciprocal of the dose required to reduce the surviving fraction to 37% (or 1/e) of its prior level for the exponential survival curve obtained at very low dose rates. The values of $\eta_{PL}$ and $\varepsilon =\frac{\varepsilon_{PL}}{\varepsilon_{2PL}}$ were determined by fitting the Co‑60 high‑dose rate C3H10T1/2 cell survival curves.^3^, ^32^ The parameter $\varepsilon_{PL}$ was selected to correspond to a characteristic mean repair time of 2 hours ($\varepsilon_{PL}=0.5\;h^{-1}$).^3^

### 2.2 Oxygen dynamics during irradiation

Intracellular oxygen availability is represented by a time‐dependent partial pressure $pO_{2}\left(t\right)$ governed by the competition between radiolytic depletion during dose delivery and recovery toward a baseline level:

(2) $$\frac{d}{dt}pO_{2}\;\left(t\right)=k_{reox}\;\left(pO_{2,0}-pO_{2}\left(t\right)\right)-G\phi \left(pO_{2}\right)\dot{D}\left(t\right)$$

Here $pO_{2,0}$ is the baseline intracellular (target‐level) oxygen partial pressure, G (mmHg/Gy) is the radiolytic depletion coefficient, and $k_{reox}$ is a reduced‐order effective recovery constant with implied time constant $\tau_{reox}=1/k_{reox}$. This formulation is intended as a compact surrogate for diffusion, vascular supply, and intracellular consumption rather than as a direct microphysiologic measurement. Consistent with physicochemical modeling of peroxyl‐radical recombination in aqueous media,^13^, ^16^ we use G=0.42 mmHg/Gy in the reference case.

We model the depletion efficiency with a Michaelis–Menten form,

(3) $$\phi \;\left(pO_{2}\right)=\frac{pO_{2}}{pO_{2}+K_{g}}$$

where $K_{g}$ is the saturation constant that suppresses further depletion at very low oxygen levels. Following prior FLASH modeling, we set $K_{g}=0.21$ mmHg (approximately 0.29μM at 37°C).^6^, ^14^, ^33^ Because $K_{g}$ is much smaller than the baseline $pO_{2}$ values explored in the physiologic window (approximately 1–5 mmHg), $\phi (pO_{2})$ remains close to unity in the reference regime.

In the ultra‐high‐dose‐rate limit relevant to FLASH, when the delivery time $\tau_{pulse}\ll \tau_{reox}$, oxygen recovery during irradiation is negligible and Equation (2) reduces to

(4) $$\frac{d}{dt}pO_{2}\approx -G\phi \left(pO_{2}\right)\dot{D}\left(t\right)$$

This reduced form clarifies that FLASH sparing requires depletion to occur on a timescale faster than effective recovery. Direct measurements of bulk oxygen during FLASH suggest that depletion is real but limited in magnitude and that tissue‐level reoxygenation occurs on the order of seconds in vivo. These observations further motivate treating $k_{\mathrm{reox}}$ as an uncertain, exploratory local parameter rather than as a fixed tissue constant.^34^, ^35^

### 2.3 Hypothesized niche‐to‐nucleus oxygen gradient

Standard radiobiological models often treat the oxygen partial pressure at the DNA target as equivalent to the bulk extracellular or vascular value. In this work, we instead treat the DNA target‐level baseline as an effective quantity that can be written schematically as

$$pO_{2}^{\mathrm{nuc}}=pO_{2}^{\mathrm{vasc}}-\Delta pO_{2}^{\mathrm{niche}}-\Delta pO_{2}^{\mathrm{mito}}$$

where $\Delta pO_{2}^{niche}$ captures extravascular diffusion and local consumption, and $\Delta pO_{2}^{mito}$ captures additional intracellular depletion associated with perinuclear oxygen consumption. This relation is intended as a mechanistic hypothesis rather than as a direct measurement of a universal cell‐by‐cell gradient.

**FIGURE 2:**
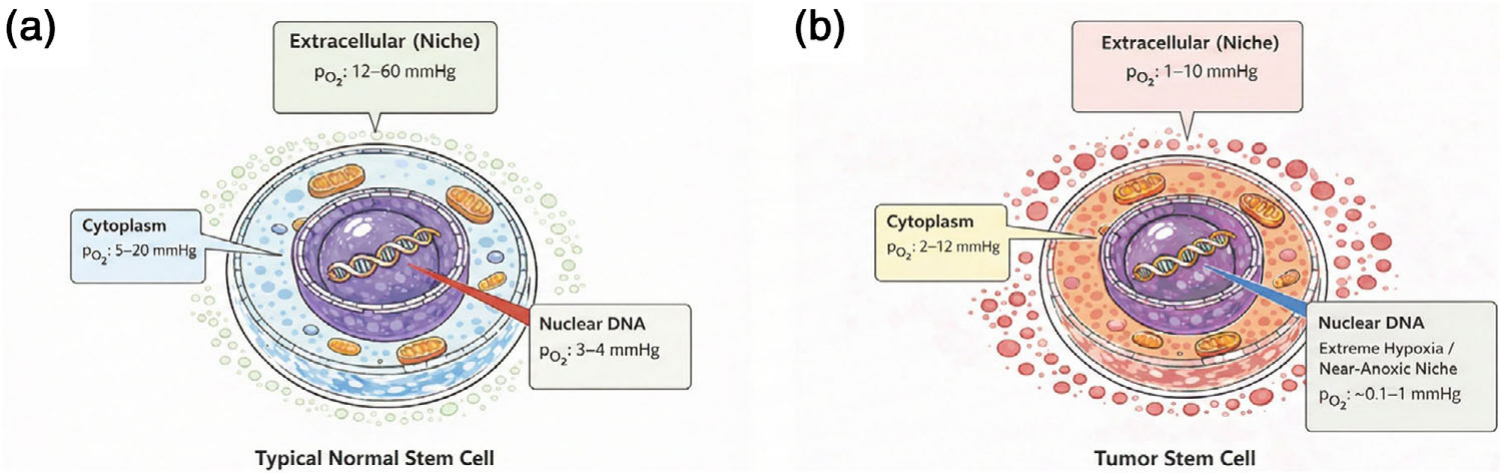
Oxygen microenvironment in normal versus tumor reference compartments (schematic, not to scale). Side‐by‐side conceptual diagrams compare illustrative oxygen partial‐pressure ranges across three representative compartments: extracellular niche, cytoplasm, and nuclear DNA. (A) A quiescent normal stem/progenitor niche with lower effective intracellular oxygenation toward the nucleus. (B) A perinecrotic tumor compartment with markedly reduced extracellular oxygenation and very low effective nuclear oxygenation. The values are intended to convey relative compartmental differences and effective target‐level baselines, not direct simultaneous in situ measurements of subcellular gradients in a single cell.

We therefore define three reference baseline regimes that differ both in oxygen tension and in biological context. For a reference quiescent normal niche, we use 3 mmHg as a hypothesis‐driven working point for effective DNA target‐level oxygenation (See Figure 2(A)). This value is lower than typical vascular marrow measurements but is intended to represent a plausible target‐level oxygenation in selected deeply quiescent stem/progenitor compartments rather than a universal tissue measurement across all normal tissues. In the adopted OER formulation, it lies near the steep part of the oxygen‐response curve and therefore within the regime most capable of showing oxygen‐mediated FLASH sparing.^23^, ^29^

For a reference perinecrotic tumor compartment, we use 0.2 mmHg to represent a radiobiological‐anoxia regime near the OER floor (See Figure 2(B)), where additional transient depletion is expected to have little further effect on fixation.^36^, ^37^ This reference state is not intended to represent all tumor clonogens or all tumor models.

For a reference well‐oxygenated perivascular comparison compartment, we use 30 mmHg to represent a regime on the OER saturation plateau, where transient depletion is expected to have negligible impact on fixation.^18^, ^21^ This comparison state is included to illustrate model behavior far from the intermediate oxygen‐sensitive window rather than to represent all perivascular microenvironments.

To support the use of nuclear oxygen rather than cell‐surface oxygen in the main lesion‐fixation model, Appendix A provides a qualitative rationale for treating the baseline DNA target–level oxygen tension ($pO_{2,0}$) as an effective quantity that can be lower than bulk vascular/extracellular oxygen in regulated stem/progenitor niches. The rationale combines (i) physiological niche hypoxia and tissue‐scale oxygen heterogeneity reported across regenerative compartments (including bone marrow, brain, intestine, and skin/hair follicle)^22^, ^23^, ^24^, ^25^, ^26^, ^27^, ^28^, ^29^, ^30^ with (ii) respiration‐dependent mitochondrial “shielding” measurements showing nuclear $pO_{2}$ can be ∼20%–40% lower than cytosolic $pO_{2}$, and that inhibiting respiration increases nuclear oxygen exposure. ^40^ Computational modeling further supports that intracellular gradients can arise under hindered diffusion and spatially localized consumption.^38^

### 2.4 Modified LPL formulation with explicit Precursor Lesion state

**FIGURE 3:**
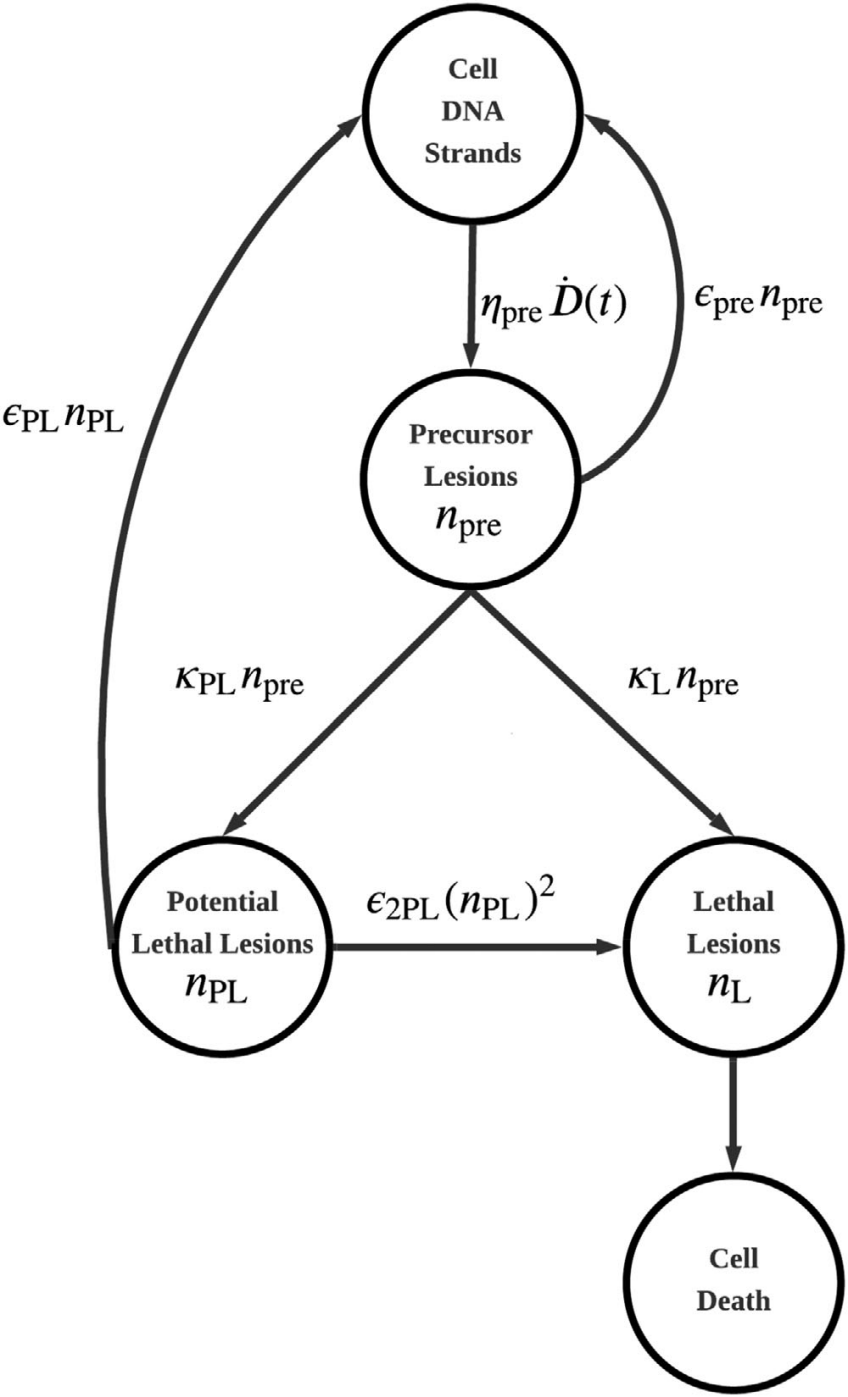
Flowchart of the Modified LPL (M‐LPL) model. The framework introduces an explicit Precursor Lesion state ($n_{pre}$) to resolve the oxygen‐dependent fixation step. Precursor Lesions are induced at rate $\eta_{pre}\dot{D}\left(t\right)$ and decay via competing pathways: fast chemical restitution ($\varepsilon_{pre}$) or fixation into potentially lethal ($\kappa_{PL}$) and lethal ($\kappa_{L}$) states. The fixation rates are functions of the instantaneous nuclear oxygen tension $pO_{2}\left(t\right)$, coupling the biological response to radiolytic oxygen depletion.

To explicitly resolve the fixation stage implicit in the classical LPL formalism, a Precursor Lesion population $n_{pre}$ is introduced. The coupled kinetic equations, written to make the competing chemical restitution and oxygen‐dependent fixation steps explicit, are (See Figure 3):

(5a) $$\begin{array}{cc} \frac{d}{dt}\;n_{pre} & =\eta_{pre}\;\dot{D}\left(t\right)-\varepsilon_{pre}n_{pre}-\kappa_{PL}\left(pO_{2}\right)n_{pre}\\ & \;-\kappa_{L}\left(pO_{2}\right)n_{pre} \end{array}$$

(5b) $$\frac{d}{dt}\;n_{PL}=\kappa_{PL}\;\left(pO_{2}\right)n_{pre}-\varepsilon_{PL}n_{PL}-\varepsilon_{2PL}{\left(n_{PL}\right)}^{2}$$

(5c) $$\frac{d}{dt}\;n_{L}=\kappa_{L}\;\left(pO_{2}\right)n_{pre}+\varepsilon_{2PL}{\left(n_{PL}\right)}^{2}$$

Following the molecular yield estimates of Ward,^2^ we set the Precursor Lesion induction rate to $\eta_{pre}\approx 2000$ $Gy^{-1}$. The chemical restitution rate constant is set to $\varepsilon_{pre}\approx 50$ $s^{-1}$, consistent with the millisecond‐scale oxygen fixation kinetics reported by Howard‐Flanders and Moore.^20^ Phenomenologically, $\eta_{pre}$ represents the aggregate yield of all ‘fixable’ radical sites—including base damage and single‐strand breaks susceptible to chemical restitution—rather than double‐strand breaks alone.

The fixation rates, $\kappa_{PL}\left(pO_{2}\right)$ and $\kappa_{L}\left(pO_{2}\right)$, are assumed to depend on the local oxygen tension. Following the oxygen‐fixation hypothesis, we assume that both rates scale proportionally with $OER(pO_{2})$. Additional justification for this choice is provided in Section 3.1.

In the numerical implementation, $\kappa_{PL}\left(pO_{2}\right)$ and $\kappa_{L}\left(pO_{2}\right)$ are chosen so that the M‐LPL model reproduces the classical LPL response under conventional dose‐rate conditions (see Equations 10a and 10b).

Oxygen modulates Precursor Lesion fixation through the local oxygen tension $pO_{2}\left(t\right)$, using the mechanistic exponential OER relationship derived by Grimes and Partridg: ^21^

(6) $$\begin{array}{cc} \mathrm{OER}\;\left(pO_{2}\right) & =OER_{\min}+\left(OER_{\max}-OER_{\min}\right)\\ & \;\cdot \left(1-e^{-\omega \;pO_{2}}\right) \end{array}$$

where ω is the oxygen‐fixation rate constant. Its reciprocal, 1/ω, is the characteristic oxygen scale; for $\omega =0.26\;\mathrm{mmH}g^{-1}$, 1/ω≈3.8 mmHg.

### 2.5 Numerical simulation strategy

The coupled system in Equations (2)–(6) is stiff because ROD occurs on microsecond–millisecond timescales, whereas biological repair evolves over minutes to hours. Irradiation is represented by a prescribed dose‐rate history $\dot{D}\left(t\right)$. For the scenarios reported here, we approximate delivery as a single continuous pulse with constant mean dose rate, $\dot{D}=D/\tau_{pulse}$, over $0<t<\tau_{pulse}$. The governing equations remain valid for explicit pulse trains, for which interpulse recovery is controlled by the ratio of gap duration to $\tau_{reox}$.

All computations were performed in Python using scipy.integrate.solve_ivp with the implicit Radau method and adaptive time stepping. Relative and absolute tolerances were set to $10^{-6}$ and $10^{-8}$, respectively. Rates originally reported in $h^{-1}$ were converted internally to $s^{-1}$. Survival fractions (SFs) were evaluated as

$$SF=\;exp\left[-n_{L}\left(\tau_{pulse}+t_{e}\right)\right],$$

where $n_{L}$ is the expected lethal‐lesion burden after allowing sufficient post‐irradiation time for transient populations to relax; throughout this work, $t_{e}=24$ h.

## 3 RESULTS

Throughout this paper, FLASH sparing is quantified as: $\Lambda =\;\ln(SF_{FLASH}/SF_{CONV})$ using a reference conventional dose rate of ∼0.05 Gy/s. For intuition, this implies $SF_{FLASH}/\;SF_{CONV}=exp\left(\Lambda \right)$; for example, Λ=0.30 corresponds to a survival ratio of exp(0.30)≈1.35 (≈ 35% higher survival under FLASH at the same total dose).

### 3.1 Recovery of classical LPL behavior and emergence of FLASH sparing

To demonstrate backward compatibility with the classical LPL formalism, we consider the regime of CONV dose rate irradiation. Under these conditions ($\dot{D}\approx 0.05$ Gy/s), the timescale of delivery a few Gy of dose, $\tau_{delivery}\geq 20\;s$, vastly exceeds the millisecond‐scale lifetime of the Precursor Lesion species ($\tau_{pre}\approx 1/\varepsilon_{pre}\approx 20\;\mathrm{ms}$). Consequently, the Precursor Lesion population $n_{pre}$ rapidly equilibrates to a quasi‐steady state where production is balanced by decay.

By setting $dn_{pre}/dt\approx 0$ in Equation (5a) and assuming Precursor Lesion removal is dominated by the fast restitution term when $\varepsilon_{pre}\gg \kappa_{PL}\;\mathrm{or}\;\kappa_{L}$, the instantaneous Precursor Lesion concentration is approximated by:

(7) $$n_{pre}\left(t\right)\approx \frac{\eta_{pre}}{\varepsilon_{pre}}\dot{D}\left(t\right)$$

In the CONV limit, the Precursor Lesion pool is slaved to the instantaneous dose rate, $n_{pre}\left(t\right)\approx \eta_{pre}\dot{D}\left(t\right)/\varepsilon_{pre}$, so fixation becomes an instantaneous “branching” of Precursor Lesions into PL and L channels. Accordingly, the model reduces to Curtis's LPL form with effective yields $\eta_{PL}\;\left(pO_{2}\right)=\kappa_{PL}\;\left(pO_{2}\right)\;\eta_{pre}/\varepsilon_{pre}$ and $\eta_{L}\;\left(pO_{2}\right)=\kappa_{L}\;\left(pO_{2}\right)\;\eta_{pre}/\varepsilon_{pre}$, making explicit that oxygen dependence enters through $\kappa_{PL}\;\mathrm{and}\;\kappa_{L}$(via OER).

**FIGURE 4:**
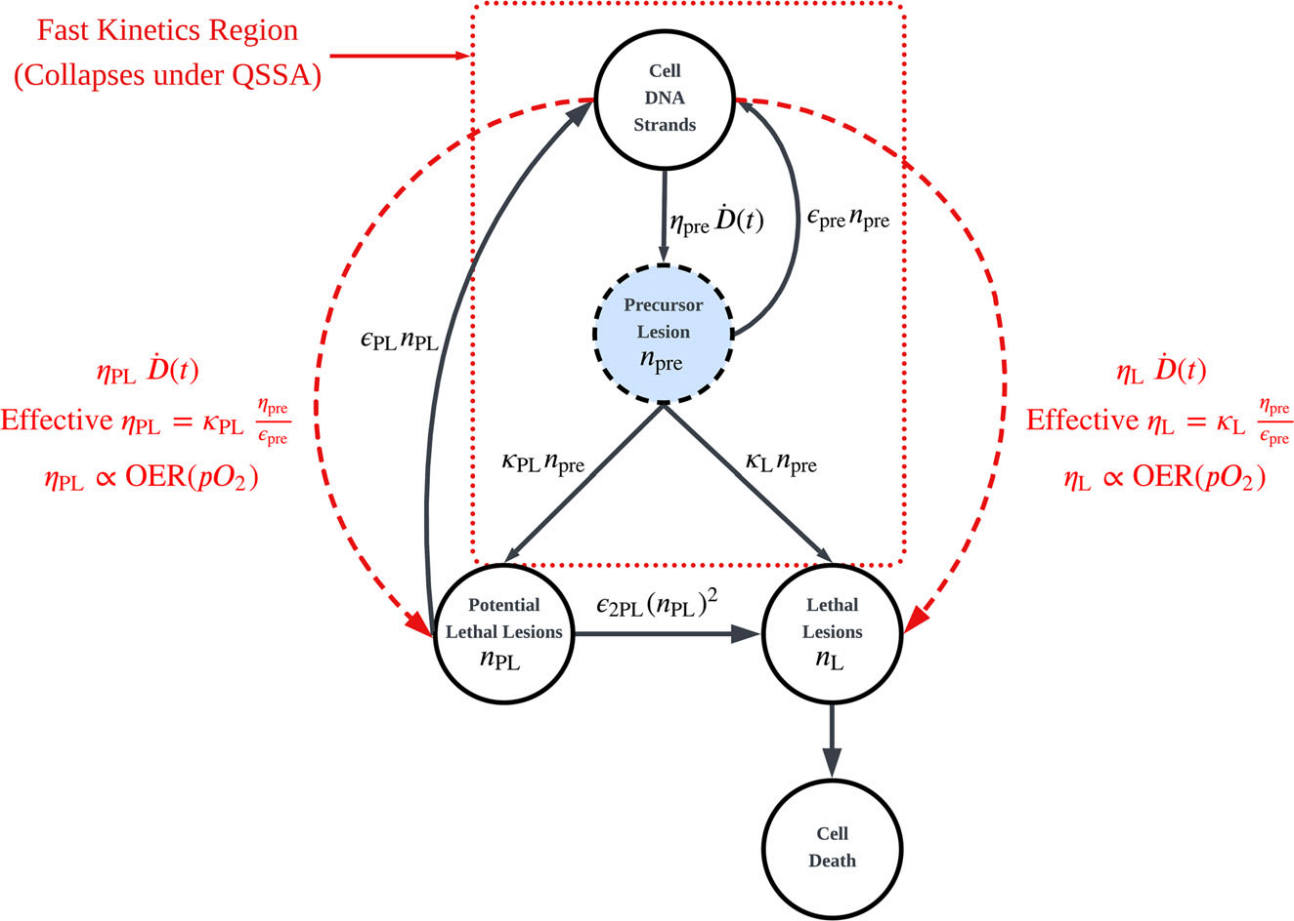
Schematic of the Quasi‐Steady State Approximation (QSSA). The diagram illustrates the timescale separation between the “Fast Kinetics Region” (red dotted box), involving Precursor Lesion population induction and restitution (∼ milliseconds), and the slower downstream processing of lesions (∼ minutes to hours). The Precursor Lesion population $n_{pre}$ is governed by competition between fast chemical restitution ($\varepsilon_{pre}$) and oxygen‐dependent fixation ($\kappa_{\mathrm{PL}}\left(pO_{2}\right)$ and $\kappa_{L}\left(pO_{2}\right)$). Under conventional dose rates ($\dot{D}\ll \varepsilon_{pre}$), $n_{pre}$ equilibrates instantaneously, mathematically reducing the explicit oxygen‐fixation steps to the effective yield parameters $\eta_{PL}$ and $\eta_{L}$ (red dashed arrows). This demonstrates that the classical LPL radiosensitivity parameters are effective quantities inherently proportional to the local Oxygen Enhancement Ratio $\left(\mathrm{OER}\left(pO_{2}\right)\right)$.

Substituting Equation (7) into the Precursor Lesion‐driven source terms in Equations (5b)–(5c) collapses the M‐LPL system to the classical LPL structure by identifying **effective (oxygen‐dependent) lesion yields**:

(8) $$\eta_{PL}^{eff}\left(pO_{2}\right)\equiv \frac{\eta_{pre}}{\varepsilon_{pre}}\kappa_{PL}\left(pO_{2}\right)$$

(9) $$\eta_{L}^{eff}\left(pO_{2}\right)\equiv \frac{\eta_{pre}}{\varepsilon_{pre}}\kappa_{L}\left(pO_{2}\right)$$

so that the production terms take the classical form $\eta_{PL}^{eff}\left(pO_{2}\right)\;\dot{D}\left(t\right)$ and $\eta_{L}^{eff}\left(pO_{2}\right)\;\dot{D}\left(t\right)$, while the downstream PL repair/misrepair sector remains unchanged.^3^ Because $\kappa_{PL}\left(pO_{2}\right)$ and $\kappa_{L}\left(pO_{2}\right)$ are modeled to scale with the instantaneous oxygen dependence (OER), this mapping makes explicit that the classical LPL radiosensitivity parameters $\eta_{PL}$ and $\eta_{L}$ are **effective quantities that inherit oxygen scaling through fixation kinetics**. In our implementation, we enforce equivalence at the conventional reference condition by selecting $\kappa_{\mathrm{PL}}\left(pO_{2}\right)$ and $\kappa_{L}\left(pO_{2}\right)$ such that $\eta_{\mathrm{PL}}^{\mathrm{eff}}\;\left(pO_{2}\right)=\eta_{\mathrm{PL}}^{\mathrm{Curt}\mathrm{is}}$ and $\eta_{L}^{\mathrm{eff}}\;\left(pO_{2}\right)=\eta_{L}^{\mathrm{Curt}\mathrm{is}}$ when $\omega pO_{2}\gg 1$ (see the equivalence relations introduced in the Methods and illustrated in Figure 4).

Furthermore, by applying the consistency requirement and using the low‑dose, long post‑irradiation repair time cell survival results reported by Curtis,^3^ our M‑LPL model predicts that the linear (α) and quadratic (β) coefficients in the LQ model satisfy

(10a) $$\alpha \;\left(pO_{2}\right)=\eta_{L}\;\left(pO_{2}\right)\propto \kappa_{L}\left(pO_{2}\right)\propto OER\left(pO_{2}\right)$$

and

(10b) $$\beta \;\left(pO_{2}\right)=\frac{{\left[\eta_{PL}\left(pO_{2}\right)\right]}^{2}}{2\varepsilon }\;\propto {\left[\kappa_{PL}\left(pO_{2}\right)\right]}^{2}\propto OER{\left(pO_{2}\right)}^{2}$$

These relationships are consistent with previous studies^36^, ^37^, ^38^ and provide the necessary confirmation of our model assumption that both fixation rates, $\kappa_{PL}\left(pO_{2}\right)$ and $\kappa_{L}\left(pO_{2}\right)$, scale proportionally with $OER(pO_{2})$. Note that $\varepsilon =\frac{\varepsilon_{PL}}{\varepsilon_{2PL}}$ is a constant that is insensitive to the local microenvironment.

**FIGURE 5:**
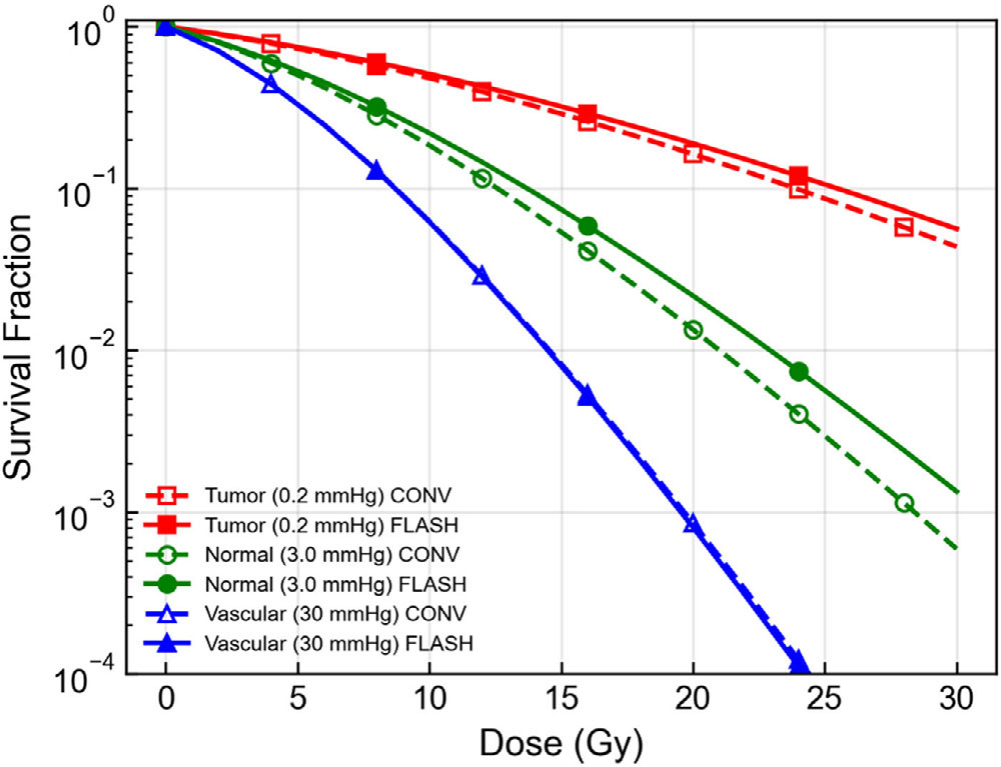
Predicted cell‐survival fractions as a function of dose for conventional (0.05 Gy/s, dashed lines) and FLASH (40 Gy/s, solid lines) irradiation. Simulations used the M‐LPL model with the Grimes exponential OER formulation. Three effective nuclear oxygen baselines were modeled: (i) a reference quiescent normal niche ($pO_{2,0}$ = 3 mmHg, green), representing a low‐mmHg transition regime in which OER remains oxygen responsive; (ii) a reference perinecrotic tumor compartment ($pO_{2,0}$ = 0.2 mmHg, red), representing near‐anoxic radiobiological conditions; and (iii) a reference perivascular compartment ($pO_{2,0}$ = 30 mmHg, blue), representing well‐oxygenated tissue. Under the reference parameter set, appreciable oxygen‐mediated sparing is observed only for the intermediate‐oxygen niche scenario, whereas the anoxic and saturated reference regimes remain nearly dose‐rate invariant.

Having established the classical limit, we simulated survival responses for the reference quiescent niche scenario, characterized by a baseline nuclear oxygen tension of $pO_{2,0}$ = 3 mmHg (Figure 5, green curves). Figure 5 illustrates that at conventional dose rates (0.05 Gy/s), the survival curve follows a standard quadratic decline. Under FLASH conditions (40 Gy/s), however, rapid ROD occurs faster than diffusional replenishment and transiently drives the effective nuclear environment toward lower oxygenation. This dynamic reduction in $pO_{2}$ suppresses Precursor Lesion fixation and increases *SF* relative to the conventional control.

**FIGURE 6:**
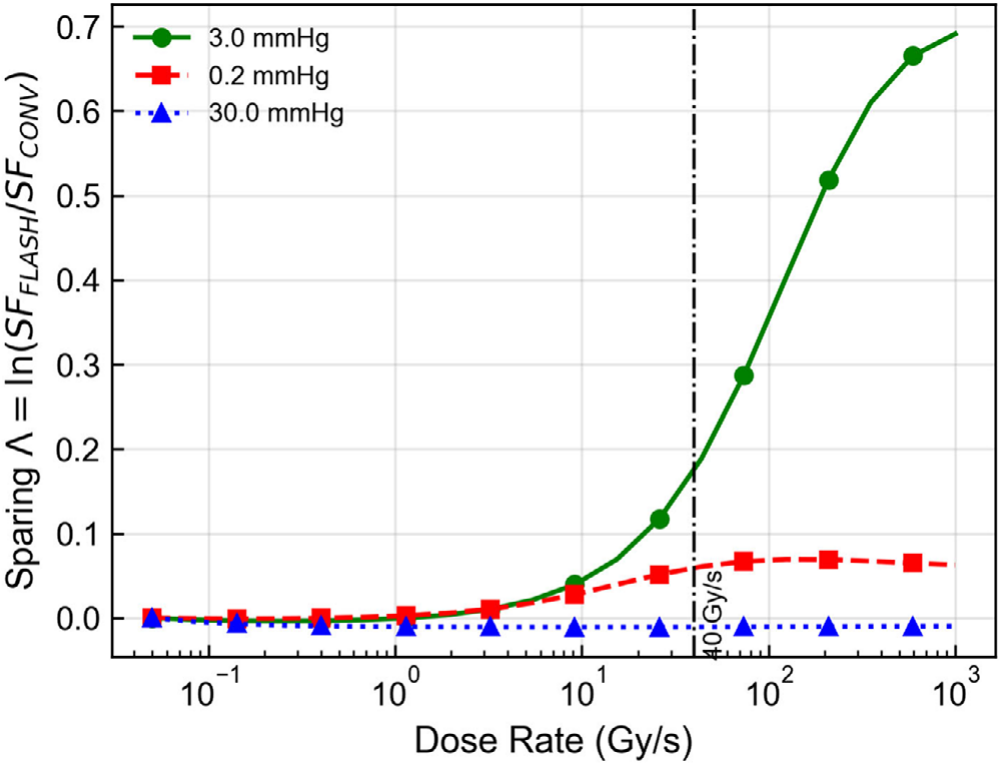
The differential sparing magnitude Λ, defined as the survival advantage of FLASH relative to conventional delivery, plotted against dose rate for a fixed dose of 10 Gy. Curves illustrate the transition from conventional to FLASH behavior for the three representative reference compartments. The quiescent normal‐niche working point (3.0 mmHg, green circles) exhibits a sharp increase in sparing as dose rate exceeds about 30 Gy/s, corresponding to the regime in which radiolytic depletion outpaces recovery. In contrast, both the perinecrotic (0.2 mmHg, red squares) and well‐oxygenated (30.0 mmHg, blue triangles) reference compartments show negligible oxygen‐mediated sparing across the explored dose‐rate range. The vertical dotted line indicates the 40 Gy/s reference point used in Figure 5.

The dose rate dependence of this sparing effect is shown in Figure 6. For the normal stem cell baseline (3.0 mmHg), the differential sparing metric $\Lambda =\;\ln(SF_{\mathrm{FLASH}}/SF_{\mathrm{CONV}})$ remains negligible at low dose rates but rises sharply as the dose rate exceeds 30–40 Gy/s, consistent with the timescales required to outpace oxygen diffusion.

### 3.2 Limited oxygen‐mediated sparing outside the OER‐sensitive window

In contrast to the reference quiescent normal niche, the tumor‐oriented reference scenarios exhibited two regimes of near invariance in Figures 5 and 6. For the perinecrotic reference compartment (red curves), modeled with baseline $pO_{2,0}$ = 0.2 mmHg, the initial state is already at the OER floor. Consequently, further radiolytic depletion produces little additional change in radiosensitivity, and the FLASH survival curve closely overlaps the conventional curve.

Similarly, for the perivascular reference compartment (blue curves, $pO_{2,0}$ = 30 mmHg), the OER is effectively saturated. While depletion still occurs, the absolute oxygen tension remains on the plateau of the OER curve, resulting in negligible changes in fixation efficiency. Figure 6 confirms that for both the 0.2 mmHg and 30 mmHg baselines, the sparing metric Λ remains small across the explored dose‐rate range.

In the well‐oxygenated limit, Λ is expected to be essentially zero; small slightly negative values can occur at high lesion burden due to the nonlinear PL–PL interaction term.

### 3.3 The physiologic‐hypoxia window

**FIGURE 7:**
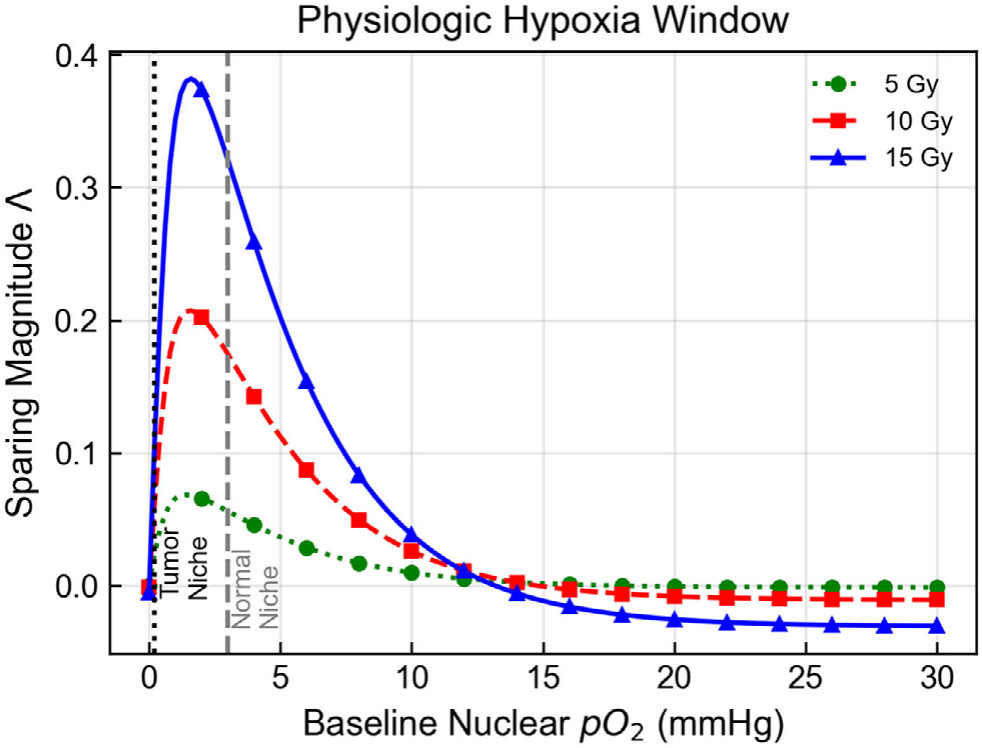
The physiologic‐hypoxia window of FLASH sparing. The differential sparing magnitude Λ is plotted as a continuous function of baseline nuclear oxygen tension $pO_{2,0}$. Curves represent total doses of 5 Gy (green, dotted), 10 Gy (red, dashed), and 15 Gy (blue, solid), delivered at a FLASH dose rate of 40 Gy/s versus a conventional reference of 0.05 Gy/s. The response follows a bell‐shaped trajectory peaking between 1 and 5 mmHg, where modest changes in $pO_{2}$ produce appreciable changes in fixation/OER. Higher doses produce greater sparing because larger total radiolytic oxygen consumption drives the system further down the OER slope. Vertical lines mark the reference quiescent normal niche (3.0 mmHg, gray), which lies near the apex of the window, and the reference perinecrotic tumor compartment (0.2 mmHg, black), which lies in the radiobiological‐anoxia region where the differential effect is suppressed.

To systematically map the differential effect, we plotted the sparing magnitude Λ as a continuous function of baseline nuclear oxygen tension for total doses of 5, 10, and 15 Gy (Figure 7). The results reveal a clear Goldilocks window of sensitivity. The sparing effect follows a bell‐shaped curve peaking between 1 and 5 mmHg, coinciding with the transition region of the OER curve where oxygen dependence remains strong. This window corresponds to the range in which $\Delta \left(\mathrm{OER}\right)/\Delta \left(pO_{2}\right)$ is largest under the adopted exponential OER model and in which clinically relevant doses can produce observable changes in survival.^31^

We observed a positive dependence on integral dose: as the dose increases from 5 to 15 Gy, the magnitude of sparing amplifies because greater total radiolytic oxygen consumption is achieved during the pulse. Notably, the reference quiescent‐niche working point (3.0 mmHg) lies near the apex of this window. In contrast, the reference perinecrotic tumor baseline (0.2 mmHg) lies in the radiobiological‐anoxia region, where the OER is effectively minimized regardless of dose.

### 3.4 Robustness to kinetic parameters

Given the uncertainty surrounding in vivo radiolytic yields and local oxygen recovery kinetics, we evaluated the sensitivity of the differential sparing effect to variations in the depletion coefficient G and the reoxygenation rate constant $k_{\mathrm{reox}}$.

**FIGURE 8:**
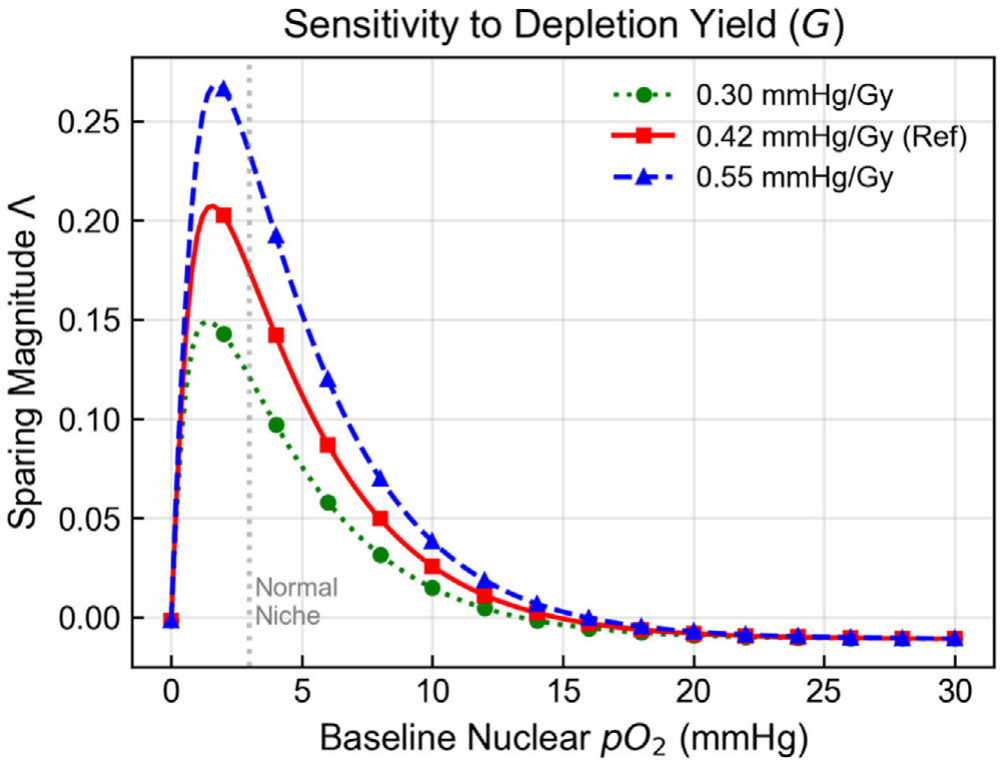
Sensitivity of the sparing effect to the radiolytic oxygen depletion coefficient G. The differential sparing magnitude Λ is simulated for a 10 Gy dose (40 Gy/s) across a range of G values. The nominal aqueous value of 0.42 mmHg/Gy (red, solid) is compared with a reduced buffered value of 0.30 mmHg/Gy (green, dotted) and a higher‐yield value of 0.55 mmHg/Gy (blue, dashed). While lower yields reduce the maximum sparing achievable, the bell‐shaped dependence on baseline $pO_{2}$ persists, confirming that the intermediate oxygen window is robust to plausible uncertainty in in vivo radical scavenging. All other parameters are adopted from Table A1.

Figure 8 illustrates the impact of varying the radiolytic depletion yield G. While the standard aqueous value is 0.42 mmHg/Gy, intracellular buffering may reduce the effective yield.^16^ Reducing G to 0.30 mmHg/Gy (green, dotted curve) suppresses the magnitude of the sparing effect Λ but does not eliminate it. The Goldilocks morphology remains intact, with the peak sensitivity centered on the physiologic niche window (1–5 mmHg). Thus, although the magnitude of FLASH sparing depends on the chemical environment, the existence of an intermediate oxygen‐sensitive window is structurally robust.

**FIGURE 9:**
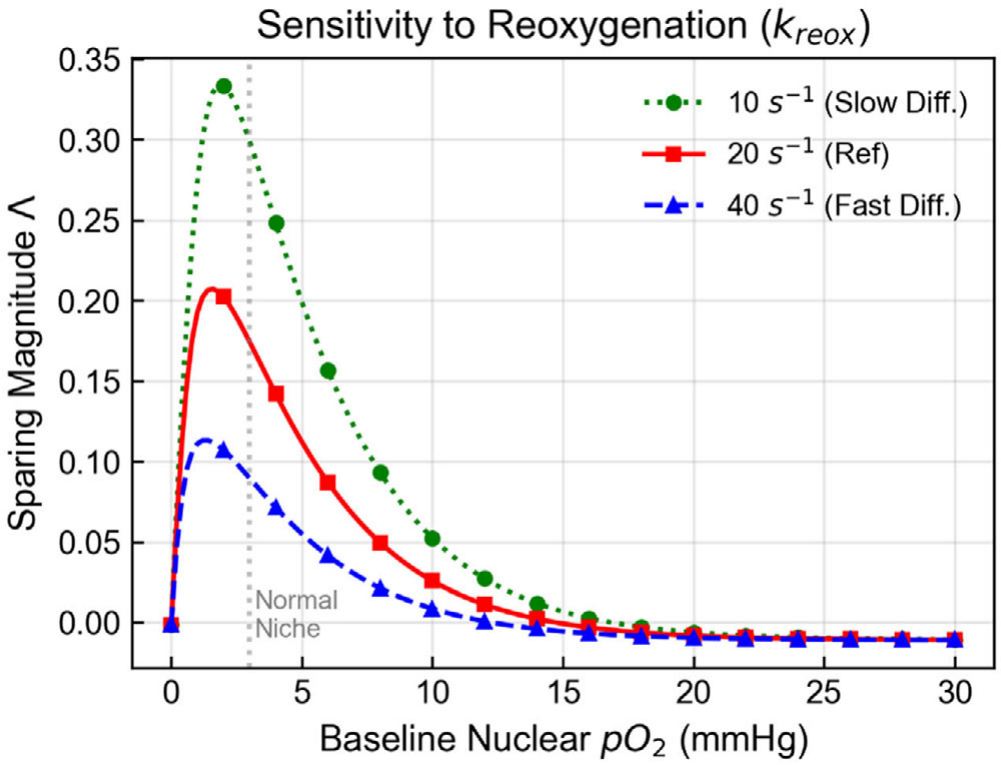
Sensitivity of the sparing effect to the reoxygenation rate constant $k_{\mathrm{reox}}$. The sparing magnitude Λ is simulated for varying $k_{\mathrm{reox}}$ values that proxy effective local oxygen recovery kinetics. The reference rate of 20 s^−1^ (red, solid) is compared with slower recovery (10 s^−1^, green, dotted) and faster recovery (40 s^−1^, blue, dashed). Sparing is maximized when recovery is slow enough for depletion to persist during the pulse, whereas faster recovery suppresses sparing. The reference 50 ms timescale implied by $k_{\mathrm{reox}}$ is interpreted here as a reduced‐order local target‐level timescale rather than as a direct bulk‐tissue reoxygenation measurement. All other parameters are adopted from Table A1.

Figure 9 examines the competition between depletion and recovery by varying $k_{\mathrm{reox}}$. The nominal rate (20 s^−1^) corresponds to an effective reference timescale of 50 ms. We emphasize that this nominal timescale is not intended to represent bulk tissue or vascular reoxygenation measured in vivo, but rather a reduced‐order local recovery timescale at the target level. Increasing $k_{\mathrm{reox}}$ to 40 s^−1^ (blue, dashed curve) significantly dampens the sparing effect because oxygen replenishment begins to outpace consumption during the pulse. Conversely, decreasing $k_{\mathrm{reox}}$ to 10 s^−1^ (green, dotted curve) enhances sparing. These results mechanistically support the requirement that delivery be completed on a timescale short relative to effective local recovery, while remaining consistent with the observation that tissue‐level oxygen recovery in vivo can occur on much slower and spatially heterogeneous timescales.^31^, ^34^, ^35^

## 4 DISCUSSION

### 4.1 Mechanistic interpretation

These results support an oxygen‐fixation interpretation of one contributor to the FLASH differential effect: a competition in time between radiolytic oxygen consumption and oxygen‐dependent chemical fixation. In the present framework, Λ is largest when baseline oxygenation lies in a regime where modest changes in $pO_{2}$ produce appreciable changes in fixation and OER. For the adopted OER curve, that sensitivity range is approximately 1–5 mmHg.

The contrast between the regulated quiescent niche working point (around 3 mmHg) and the tumor‐oriented reference states explored here should therefore be interpreted only as a compartment‐level hypothesis, not as a universal binary distinction between normal tissue and tumor response. What the model predicts is not a universal tissue rule, but a parameter regime: oxygen‐mediated sparing should be strongest when effective target‐level oxygenation begins near the steep portion of the OER curve.

### 4.2 Anatomical concordance

A useful qualitative consistency check is the anatomical pattern of reported FLASH sparing. Organs in which toxicity depends on spatially organized stem/progenitor compartments — including settings with acute epithelial injury as well as later regenerative failure — are among those showing reproducible sparing, such as skin, intestine, and brain, including the whole‐brain FLASH study of Montay‐Gruel et al.^28^ These are also tissues in which physiological hypoxia and stem‐cell niche organization are biologically relevant.^22^, ^23^, ^29^

This anatomical concordance does not prove the model, but it strengthens the biological plausibility of using regulated regenerative compartments as the reference context for oxygen‐mediated sparing.

### 4.3 Constraints on dose rate: The diffusion limit

The model also clarifies why a dose‐rate threshold is expected: depletion must be established on a timescale comparable to, or shorter than, effective recovery. For a pulse of dose D delivered over time *T*, appreciable sparing requires $T≲\tau_{reox}$. This prediction is consistent with prior dimensional analyses and with quantitative dose‐threshold arguments for FLASH normal‐tissue sparing.^25^, ^31^

### 4.4 Tumor heterogeneity and regime dependence

An important concern is whether the same oxygen‐mediated mechanism could also influence tumor subpopulations that transiently reside near the intermediate oxygen‐sensitive window. The illustrative tumor‐oriented reference regimes explored here do not exhibit substantial oxygen‐mediated sparing, but these reference states are not intended to exhaust the biological heterogeneity of tumors.

This limitation is especially important for transplanted mouse xenograft models derived from immortalized cell lines, in which the classical concept of a small, spatially restricted clonogenic subpopulation may be less appropriate than in structured human tumors. More generally, tumor oxygenation is heterogeneous and dynamic, and tumor response to FLASH cannot be reduced to a single baseline oxygen state.

Consistent with this caution, Leavitt et al. reported in several subcutaneous xenograft models that acute hypoxia induced by vascular clamping did not diminish FLASH antitumor efficacy relative to conventional irradiation; rather, FLASH retained efficacy in this radiation‐resistant context.^39^ These findings indicate that acute hypoxia alone does not define a universal tumor‐response rule for FLASH and that additional biological mechanisms and tumor‐specific contexts must be considered.

An additional, still‐speculative possibility is that effective nuclear oxygen shielding may differ between normal regenerative compartments and tumor cells because of differences in metabolic phenotype. If mitochondrial oxygen consumption contributes to lowering oxygen exposure at the DNA target, then tumor‐associated metabolic reprogramming could alter the effective baseline nuclear oxygenation relative to that of quiescent normal stem cell niches. In the present model, such differences would be absorbed into the effective target‐level baseline oxygen parameter rather than treated as an independently validated mechanism. The present M‐LPL framework should therefore be interpreted as a regime‐dependent, mechanistic model for one possible contributor to oxygen‐mediated FLASH sparing in regulated normal regenerative compartments, rather than as a comprehensive or universal model of tumor response.

### 4.5 Limitations and future directions

First, oxygen kinetics are represented using a lumped recovery constant $k_{\mathrm{reox}}$. While this reduced‐order approach captures the dominant competition between depletion and recovery, a natural extension would be a spatially resolved reaction–diffusion formulation that explicitly incorporates vascular geometry, diffusion length, and compartment‐specific oxygen consumption. The nominal 50 ms recovery timescale associated with the reference $k_{\mathrm{reox}}$ should be interpreted as an effective local target‐level timescale rather than as a direct in vivo tissue or vascular reoxygenation measurement. This conservative treatment is consistent with direct measurements showing that bulk oxygen depletion during FLASH is real but limited in magnitude and that tissue oxygen can rebound on the order of seconds in vivo.^34^, ^35^

Second, several parameters remain uncertain, particularly the in situ depletion coefficient G, the effective recovery rate $k_{reox}$, and the mapping from extracellular to DNA target‐level oxygenation. Larger uncertainty would be expected to shift the amplitude and threshold of sparing more strongly than the existence of the intermediate oxygen‐sensitive window itself.

Third, Appendix A provides a qualitative rationale for the low‐mmHg baseline nuclear oxygen working point used in the reference quiescent‐niche scenario. Rather than interpreting $pO_{2,0}$ = 3 mmHg as a universal tissue measurement, we treat it as a severe‐microdomain hypothesis that can arise from the combination of physiological niche hypoxia/tissue‐scale heterogeneity and respiration‐mediated nuclear oxygen shielding.^22^, ^23^, ^24^, ^25^, ^26^, ^27^, ^29^, ^30^, ^40^

Finally, oxygen‐dependent fixation may not be the only contributor to FLASH. Physicochemical mechanisms involving radical‐radical recombination, redox metabolism, immune modulation, or differential mitochondrial responses could act synergistically with or independently of oxygen depletion.^10^, ^11^, ^16^, ^17^, ^33^

Additionally, we report results using a constant‐mean‐dose‐rate representation of delivery. Explicit modeling of accelerator‐specific pulse trains may reduce, preserve, or occasionally accentuate depletion depending on interpulse spacing relative to $\tau_{reox}$, although the present ODE framework can accommodate arbitrary dose‐rate histories.

## 5 CONCLUSIONS

We developed a mechanistic extension of the LPL model that explicitly resolves Precursor Lesion induction, oxygen‐dependent fixation, and restitution/repair. By coupling fixation kinetics to a time‐dependent nuclear oxygen partial pressure governed by radiolytic depletion and reduced‐order recovery, the model predicts that oxygen‐mediated FLASH sparing is most likely when effective baseline oxygenation lies in an intermediate physiologic‐hypoxia window near 1–5 mmHg. In the reference parameter set, a quiescent normal niche working point at 3 mmHg falls near this window, whereas the illustrative low‐oxygen and high‐oxygen tumor‐oriented reference states do not. More generally, the M‐LPL framework is regime dependent rather than universal: oxygen‐mediated sparing is favored when effective baseline oxygenation lies within an intermediate OER‐sensitive window, whereas outside that window the model predicts little oxygen‐mediated differential effect. Tumor response is expected to remain heterogeneous and likely influenced by additional mechanisms beyond the present oxygen‐fixation formulation. The present work should therefore be interpreted as a mechanistic, falsifiable framework that predicts specific dependencies on baseline oxygenation, dose rate, total dose, and pulse structure, rather than as a quantitatively validated universal explanation of all FLASH responses.

## CONFLICT OF INTEREST STATEMENT

The author declares no conflicts of interest.

## FUNDING INFORMATION

The project described is supported by the National Institute of General Medical Sciences, U54 GM115458, which funds the Great Plains IDeA‐CTR Network. The content is solely the responsibility of the authors and does not necessarily represent the official views of the NIH.

## APPENDIX A NICHE‐TO‐NUCLEUS OXYGEN RATIONALE AND SENSITIVITY ANALYSIS

### Rationale for using low‐mmHg nuclear $pO_{2}$ in normal stem cell niches

To clarify the plausibility of a low baseline DNA target–level oxygen tension, we treat the baseline nuclear $pO_{2}$ ($pO_{2,0}$) as an effective variable that can be lower than bulk vascular/extracellular values in regulated stem/progenitor niches. The intent is not to claim a single universal nuclear $pO_{2}$ across tissues, but to motivate a conservative lower‐tail working point for deeply quiescent niches in which oxygen remains on the steep, oxygen‐responsive portion of the OER curve.

Two mechanisms motivate this effective low‐mmHg nuclear baseline. First, physiological oxygenation is spatially heterogeneous across normal tissues, and multiple regenerative compartments (e.g., bone marrow Hematopietic Stem Cell (HSC) regions, neural neurogenic niches, intestinal mucosa along the crypt–villus axis, and skin/hair follicle compartments) are described as operating in physiological hypoxia compared with atmospheric oxygen.^22^, ^23^, ^24^, ^25^, ^26^, ^27^, ^29^, ^30^ Second, recent subcellular oxygen imaging demonstrates a respiration‐dependent mitochondrial “shielding” effect in which nuclear $pO_{2}$ is approximately 20%–40% lower than cytosolic $pO_{2}$ under imposed oxygen levels, and inhibiting mitochondrial respiration increases nuclear oxygen exposure.^40^

Importantly, the possibility of a non‐negligible nucleus‐to‐cytosol oxygen offset is also supported by computational modeling and imaging studies showing that intracellular oxygen gradients can emerge when diffusion is effectively hindered and/or oxygen consumption is spatially localized.^38^ Together, tissue‐scale oxygen heterogeneity plus respiration‐mediated nuclear shielding provides a mechanistic rationale for exploring $pO_{2,0}$ ≈ 3 mmHg as a plausible severe‐microdomain case for selected quiescent stem‐cell niches.

In the main text, this 3‐mmHg value is therefore used as a hypothesis‐driven reference point (near the peak sensitivity window of the OER curve), while the model's qualitative predictions are tested by sensitivity analyses across a wider physiologic‐hypoxia envelope (approximately 1–5 mmHg) and by comparison with anoxic and well‐oxygenated reference regimes.

After the original submission of this work, Litzenberg published a mechanistic analysis proposing that subcellular oxygen transport and perinuclear mitochondrial organization may reduce nuclear oxygen relative to cytosolic or bulk tissue oxygen. That later work is qualitatively consistent with our use of $pO_{2,0}$ = 3 mmHg as an effective target‐level nuclear working point in selected severe microdomains. However, our adoption of 3 mmHg in the present model was made independently in the original submission as a hypothesis‐driven, non‐universal reference value and was not derived from that later paper.^41^

**TABLE A1:** Key M‐LPL model parameters.

Symbol			
M‐LPL parameters	Parameter definition	Value	Source/Rationale
$\eta_{\mathrm{pre}}$	Precursor Lesion creation rate	≈2000 $Gy^{-1}$	Ward molecular‐yield estimate^2^
$\varepsilon_{\mathrm{pre}}$	Precursor repair rate	≈50 $s^{-1}$	Millisecond‐scale oxygen‐fixation kinetics^20^
G	Radiolytic depletion coefficient	0.42 mmHg/Gy	Reference aqueous/ROD‐style value^13^
$k_{\mathrm{reox}}$	Reoxygenation rate constant	20 $s^{-1}$	Nominal effective local recovery constant (50 ms), exploratory target‐level timescale^14^, ^34^, ^35^
$K_{g}$	Depletion saturation constant	0.21 mmHg	Prior FLASH modeling; low‐$O_{2}$ saturation constant^14^
$pO_{2,0}^{Normal}$	Baseline nuclear $p_{O_{2}}$	3.0 mmHg	Hypothesis‐driven quiescent niche working point; physiological niche hypoxia/normoxia^23^, ^29^, ^30^ plus nuclear shielding / intracellular gradients^38^, ^40^
$pO_{2,0}^{Tumor}$	Baseline nuclear $p_{O_{2}}$	0.2 mmHg	Radiobiological‐anoxia/perinecrotic reference state^36^, ^37^
	Baseline nuclear	30 mmHg	Well‐oxygenated comparison state on the OER saturation plateau^18^, ^21^
$OER_{\max}$	Max. oxygen enhancement ratio	3.0	Hall and Giaccia / classical OER ceiling^1^, ^18^
$OER_{\min}$	Min. oxygen enhancement ratio	1.0	By definition
ω	OER steepness parameter	0.26 $\mathrm{mmH}g^{-1}$	Grimes mechanistic OER steepness^21^
**Classical LPL**			
$\eta_{L}$	Lethal lesion production	0.137 $Gy^{-1}$	Curtis fits to C3H10T1/2 survival data^3^, ^32^
$\eta_{PL}$	Potentially lethal production	0.6 $Gy^{-1}$	Curtis fits to C3H10T1/2 survival data^3^, ^32^
$\varepsilon_{PL}$	Repair rate	0.5 $h^{-1}$	Characteristic mean cell repair time^3^
$\varepsilon_{2PL}$	Misrepair rate	0.056 $h^{-1}$	Curtis fits to C3H10T1/2 survival data^3^, ^32^

### Local parameter sensitivity analysis

To assess the robustness of the predicted FLASH sparing, we quantified the local sensitivity of the differential sparing metric Λ to small perturbations of each model parameter about its nominal value in Table A1.

For each condition in the grid (baseline $pO_{2}$ = 0.2, 3, 30 mmHg; total dose D = 5, 10, 15 Gy; FLASH dose rate = 30, 40, 50 Gy/s), the M‐LPL Ordinary Differential Equation (ODE) system was solved using the stiff, adaptive, error‐controlled ODE solver described in the main Methods (implicit Radau integrator). Irradiation was modeled as a constant dose rate from t=0 to T=D/(doserate), followed by dose rate = 0 during the post‐irradiation period. Survival was assessed 24 h after irradiation as $SF\;\left(t\right)=exp\left[-n_{L}\left(t\right)\right]$, where $n_{L}$ is the accumulated lethal‐lesion burden.

For a parameter θ, we used a symmetric relative perturbation while holding all other parameters fixed and defined the dimensionless log‐sensitivity index $S_{\theta }\equiv \left|d\Lambda /d\;ln\;\theta \right|$. Define symmetric perturbations: $\theta_{\pm }=\theta_{0}\;\left(1\pm \delta \right)$, then numerically,

$$S_{\theta }\approx \left|\frac{\Lambda \left(\theta_{+}\right)-\Lambda \left(\theta_{-}\right)}{\ln\left(\left(\theta_{+}\right)\right)-ln\left(\left(\theta_{-}\right)\right)}\right|$$

with δ=0.10.

Table A2 summarizes the mean absolute sensitivity $|S_{\theta }|$ for key parameters, averaged over the dose and FLASH dose‐rate grid at each baseline $pO_{2,0}$. We emphasize absolute sensitivities because Λ approaches zero in the well‐oxygenated limit, which can make relative normalized metrics numerically unstable.

Across the 27‐condition grid, Λ exhibited a strong oxygen dependence. At $pO_{2,0}$ = 3 mmHg (physiologic niche), Λ ranged from 0.042 to 0.327 and increased monotonically with both dose and FLASH dose rate. At $pO_{2,0}$ = 0.2 mmHg (anoxic reference tumor), Λ was smaller (0.020–0.098), reflecting that the response is already near the OER floor. At $pO_{2,0}$ = 30 mmHg (well oxygenated), Λ was near zero and slightly negative across the explored space (−0.020 to −0.001), consistent with a small contribution from the nonlinear PL–PL interaction term under high lesion burden.

The dominant drivers of Λ depended on baseline oxygenation. In the physiologic‐hypoxia window (3 mmHg), Λ was most sensitive to oxygen kinetics—particularly the radiolytic depletion yield G and the reoxygenation rate $k_{\mathrm{reox}}$—because these parameters control the extent and recovery of transient depletion during irradiation. In contrast, under anoxic conditions (0.2 mmHg), the leading sensitivities shifted toward parameters governing the OER‐curve curvature (ω) and the lesion‐yield mapping, since additional depletion produces limited further reduction in fixation. In the well‐oxygenated regime (30 mmHg), sensitivities were dominated by the PL repair/misrepair sector, while oxygen‐kinetic parameters became negligible, consistent with saturation of the OER curve. Parameters such as G, $k_{\mathrm{reox}}$, and the effective baseline $pO_{2,0}$ are also the ones most likely to vary by more than ± 10% across tissues, so broader uncertainty is expected primarily to shift the amplitude and threshold of the effect rather than abolish the window itself.

**TABLE A2:** Mean absolute local sensitivity |dΛ/dlnθ| by baseline oxygenation.

Parameter	Definition	Perinecrotic (0.2 mmHg)	Normal niche (3 mmHg)	Perivascular (30 mmHg)
G	Radiolytic oxygen depletion yield	0.018	0.17	1.90e‐04
$k_{reox}$	Reoxygenation rate	0.017	0.141	1.58e‐04
ω	OER curve steepness (exponential model)	0.057	0.062	0.001
OERmax	Maximum OER	0.018	0.073	8.89e‐05
$\eta_{PL}^{ref}$	Potentially lethal lesion yield (Curtis mapping)	0.037	0.116	0.013
$\eta_{L}^{ref}$	Lethal lesion yield (Curtis mapping)	0.033	0.076	7.44e‐05
$\varepsilon_{PL}$	PL lesion repair rate	0.017	0.044	0.006
$\varepsilon_{2PL}$	Quadratic misrepair coefficient	0.015	0.038	0.004
